# Supplementary figures and images for: Aicardi-Goutières syndrome gene Rnaseh2c is a metastasis susceptibility gene in breast cancer
Source: PLoS Genet. 2019 May 24;15(5):e1008020. doi: 10.1371/journal.pgen.1008020 (PMC6553800; doi:10.1371/journal.pgen.1008020)

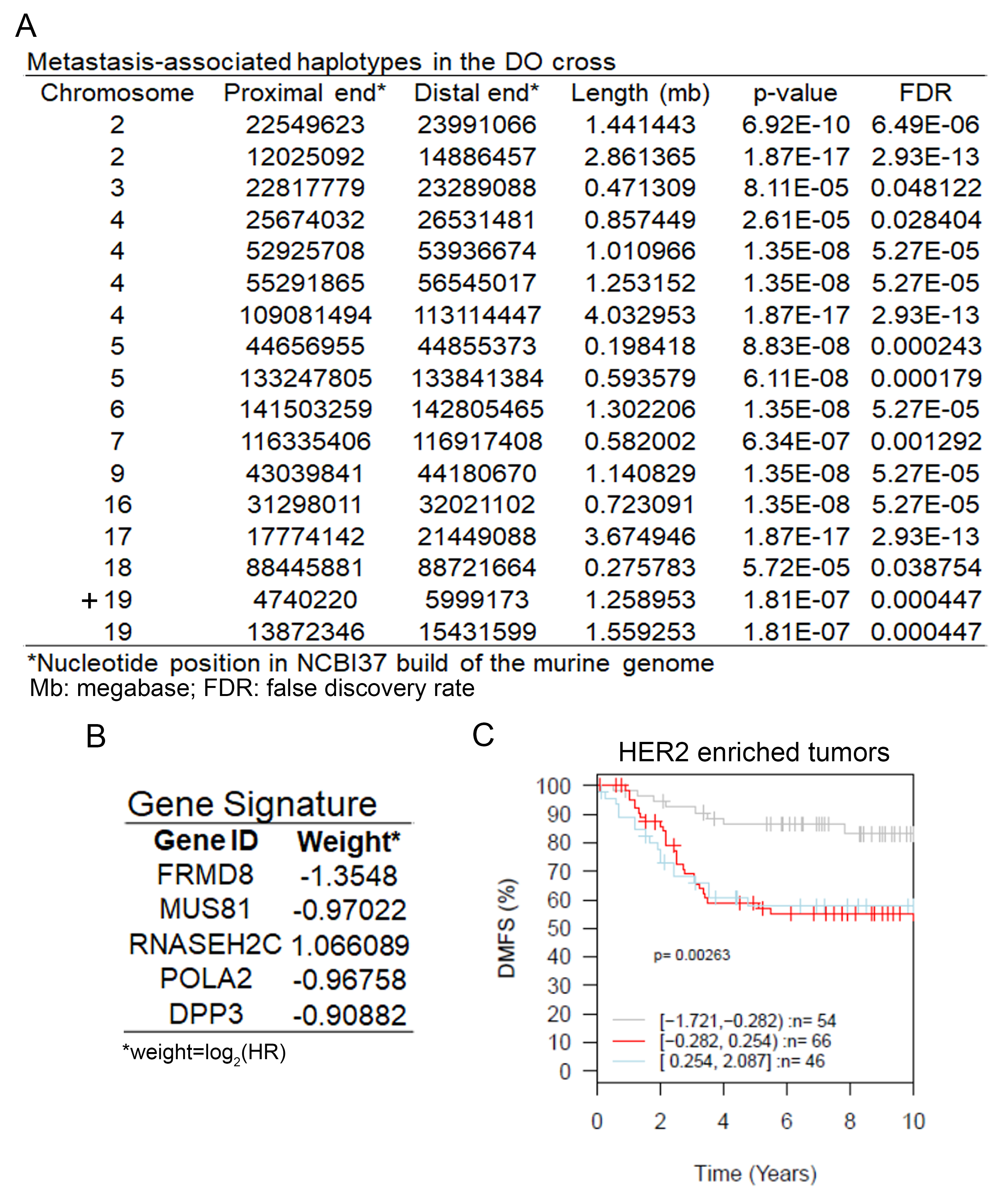

Supplement: S1 Fig — (A) Metastasis-associated haplotypes identified using the DO x PyMT cross described in Fig 1A. Plus sign denotes the haplotype selected for further investigation. (B) 5-gene signature of the chromosome 19 haplotype selected in (A) and their weights calculated using the mouse hazard ratios. (C) Kaplan-Meier analysis of distant metastasis-free survival (DMFS) of patients from the GOBO dataset stratified into terciles of the cumulative signature score of the gene signature in (B). Scores are calculated in GOBO by considering both the direction of expression and relative contribution of the gene to the signature as represented by the signature weight. HR-hazard ratio. (TIF) [file pgen.1008020.s001.tif]

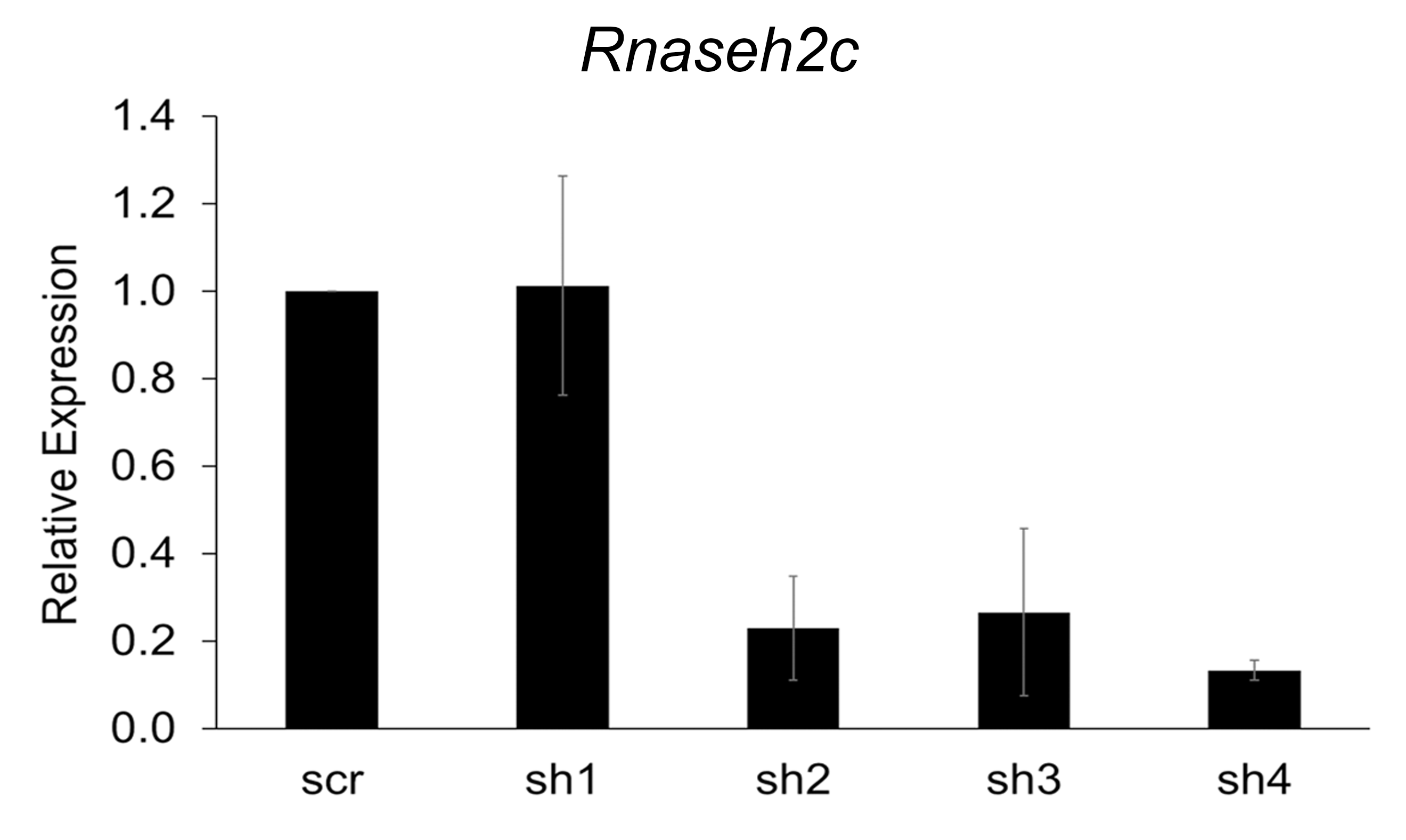

Supplement: S2 Fig — qRT-PCR analysis of Rnaseh2c expression following shRNA-mediated knockdown in Mvt1 cells. Average ± standard error of three experiments. (TIF) [file pgen.1008020.s002.tif]

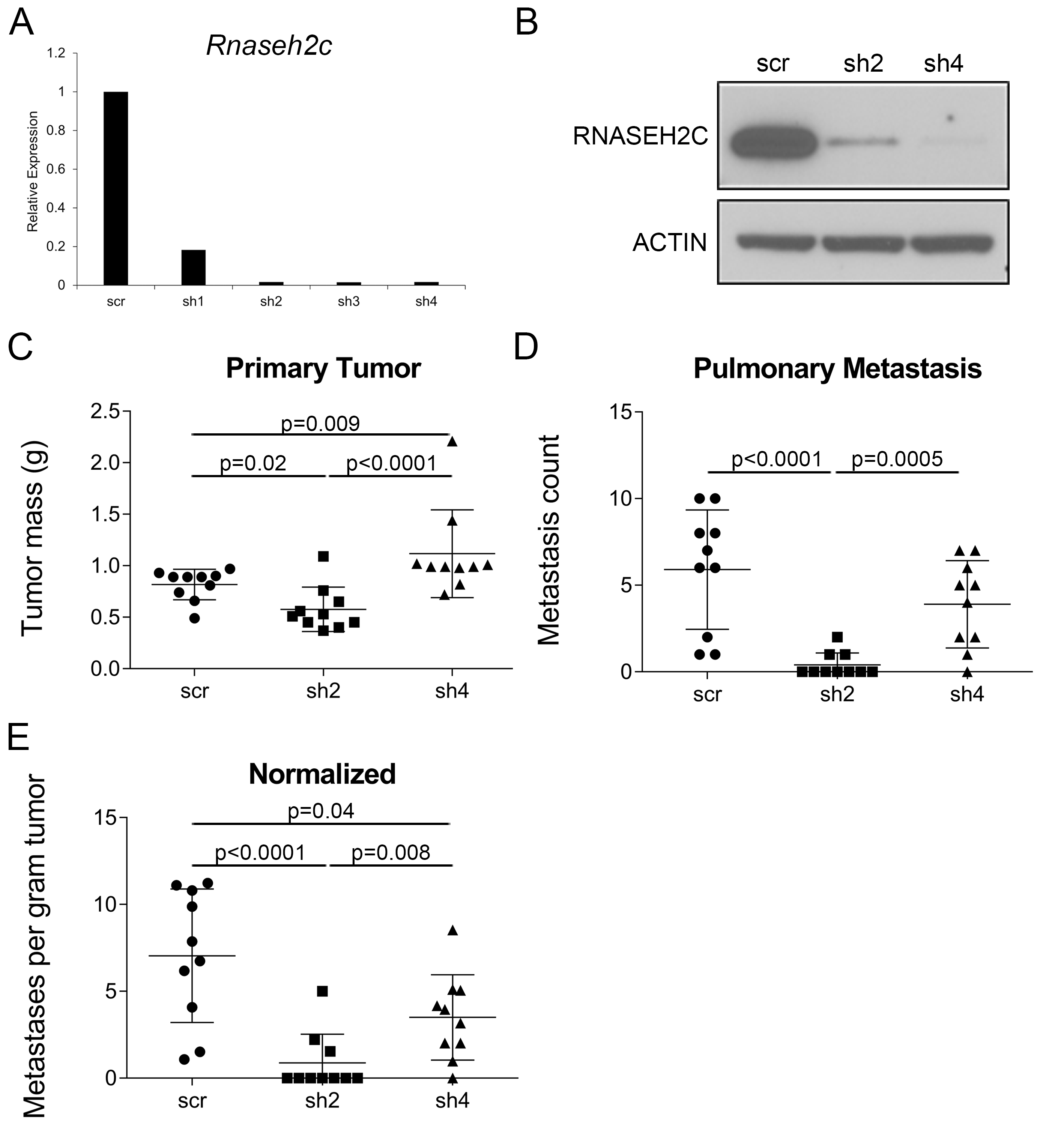

Supplement: S3 Fig — (A) qRT-PCR analysis of Rnaseh2c expression following shRNA-mediated knockdown in 4T1 cells. (B) RNASEH2C protein expression by western blot. One representative experiment is shown. (C-E) Spontaneous metastasis of 4T1 knockdown lines sh2 and sh4 was assessed as described. Tumor mass (C) and pulmonary metastases (D) were quantified at euthanasia and normalized (metastases per gram of tumor, E); average ± standard deviation, n = 10 mice per group. (TIF) [file pgen.1008020.s003.tif]

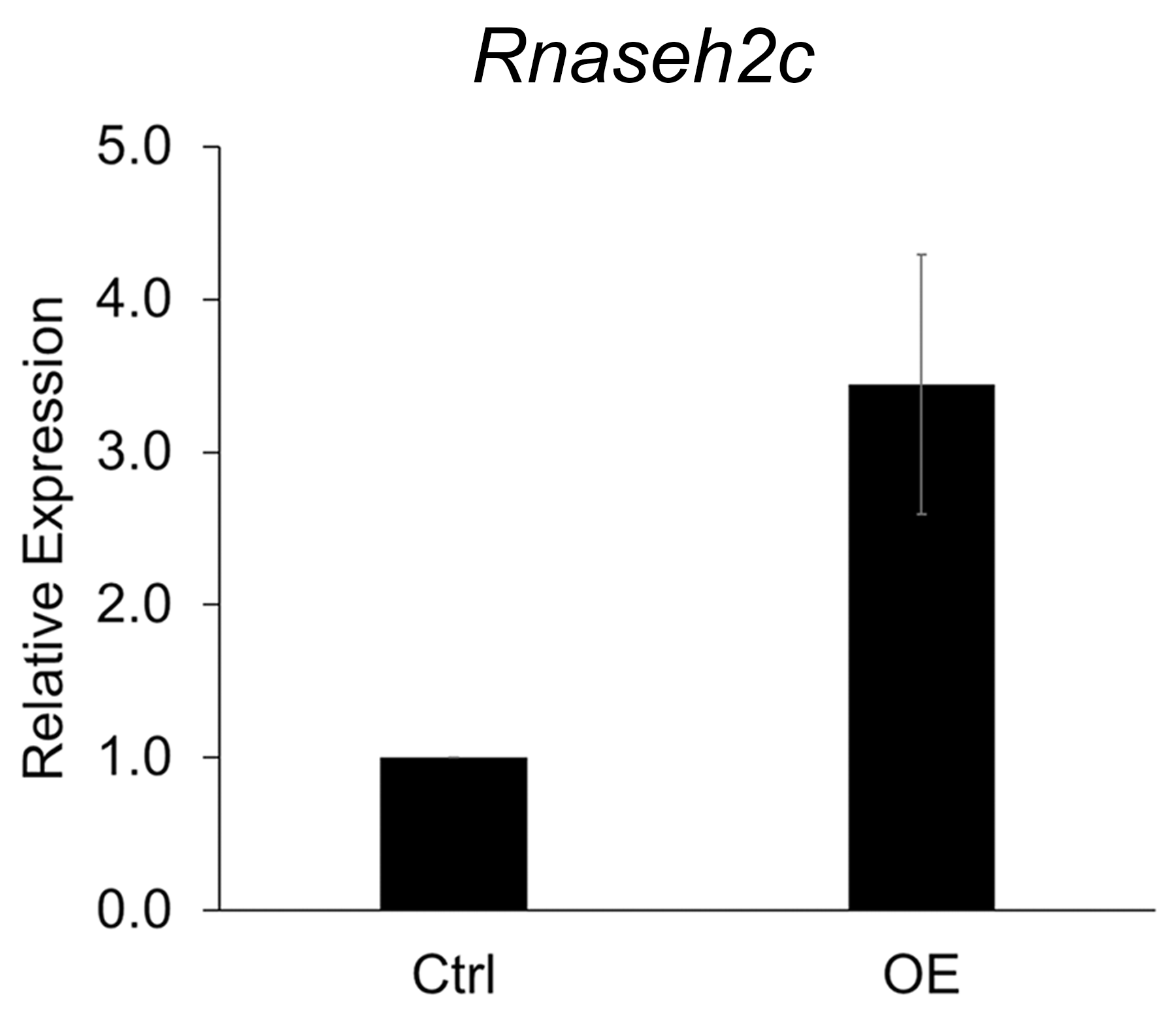

Supplement: S4 Fig — qRT-PCR analysis of Rnaseh2c expression following transduction of Mvt1 cells with an exogenous expression construct. Average ± standard deviation of three experiments. (TIF) [file pgen.1008020.s004.tif]

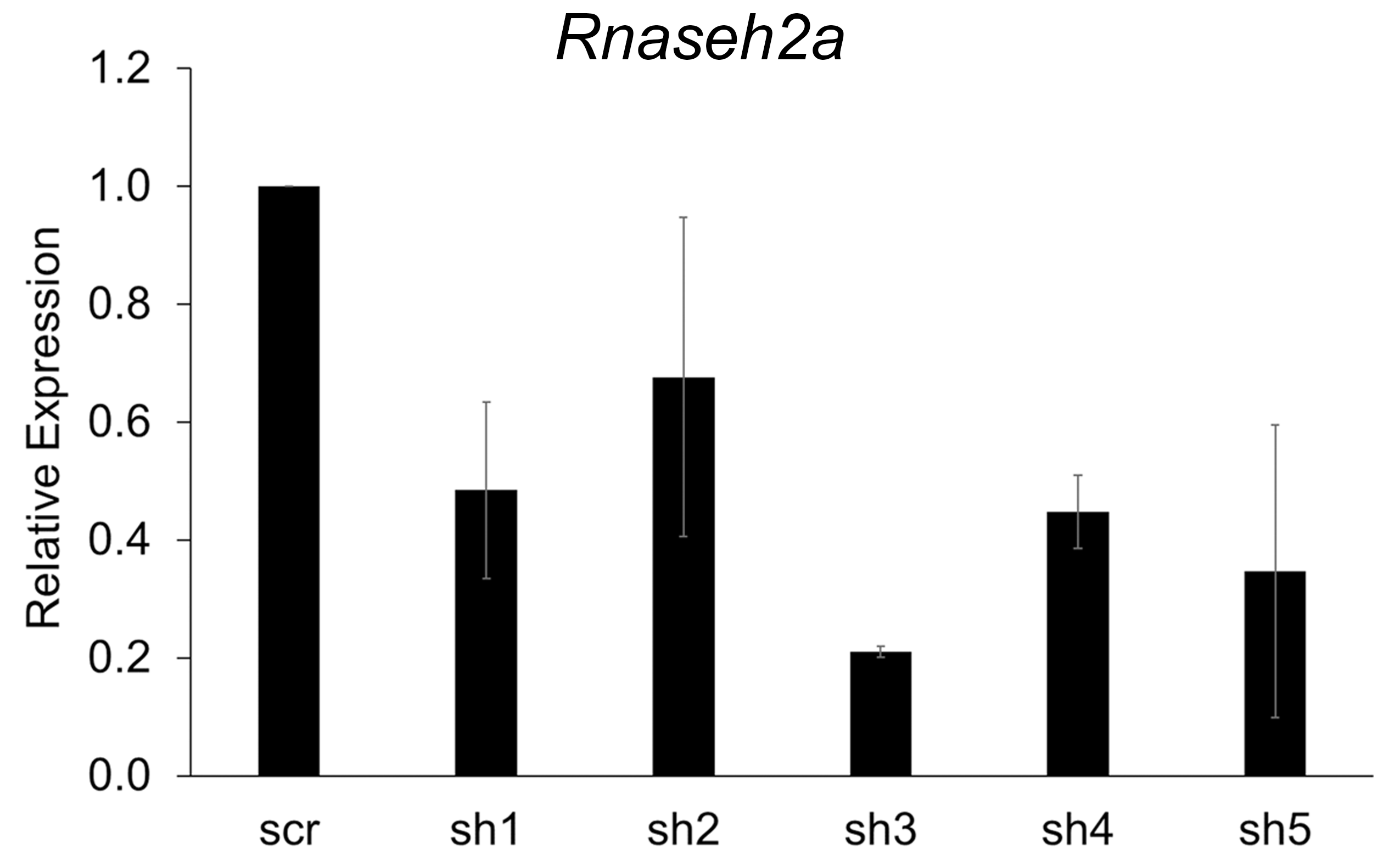

Supplement: S5 Fig — qRT-PCR analysis of Rnaseh2a expression following shRNA-mediated knockdown in Mvt1 cells. Average ± standard deviation of three experiments. (TIF) [file pgen.1008020.s005.tif]

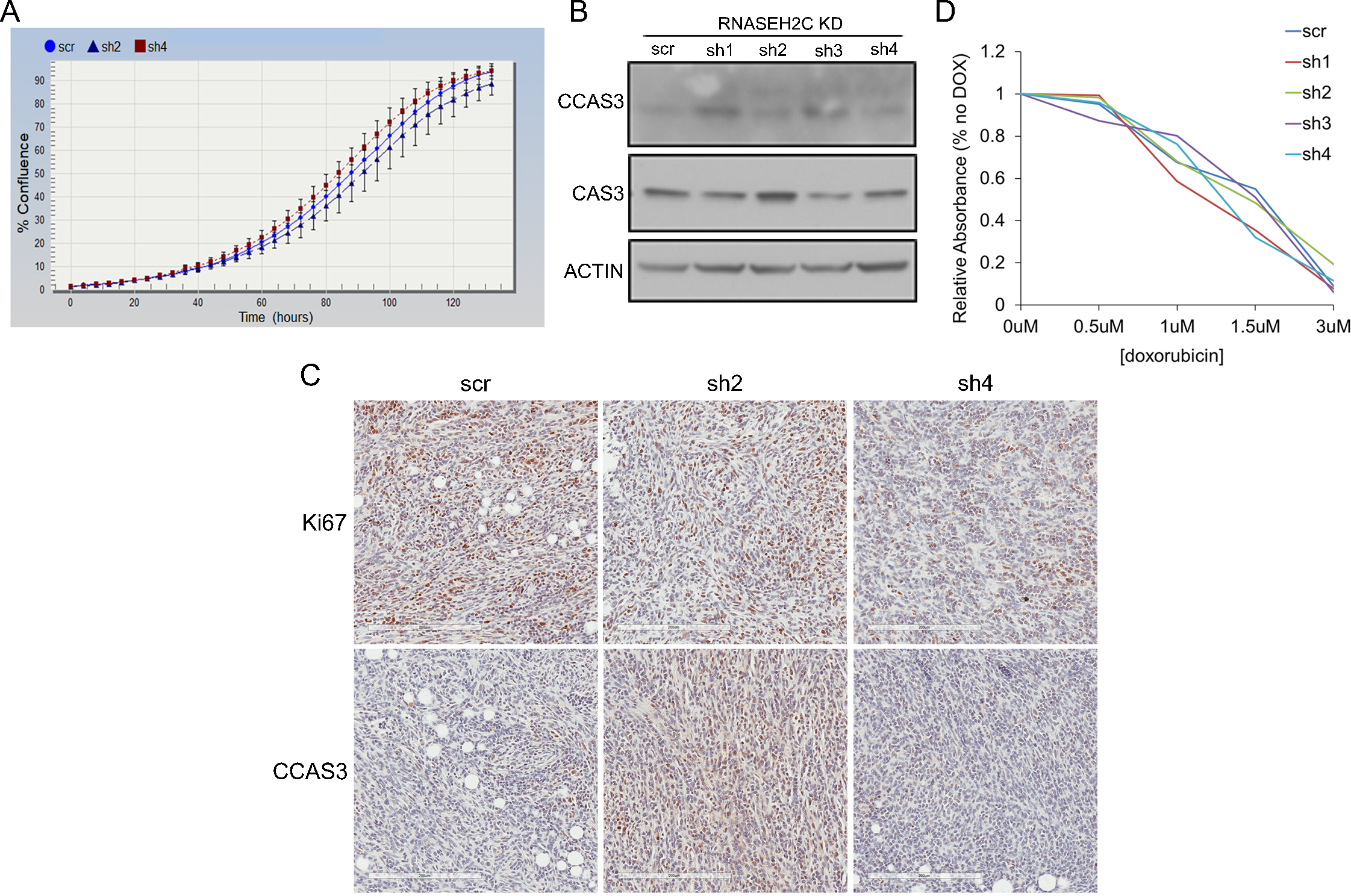

Supplement: S6 Fig — (A) In vitro cellular confluence was monitored as an indirect measurement of proliferation using the IncuCyte imaging system; average ± standard deviation of six technical replicates. (B) Full length and cleaved caspase 3 analysis in Rnaseh2c knockdown cells by western blot. (C) Ki67 (top) and cleaved caspase 3 (bottom) staining by IHC of tumor sections, representative image of staining three independent tumors. Quantification is shown in Fig 3G. (D) Rnaseh2c knockdown cells were treated with increasing concentrations of doxorubicin over 24 hours and cell viability was measured using the MTT assay. Absorbance at 570nm is reported as a percentage of the untreated condition. (TIF) [file pgen.1008020.s006.tif]

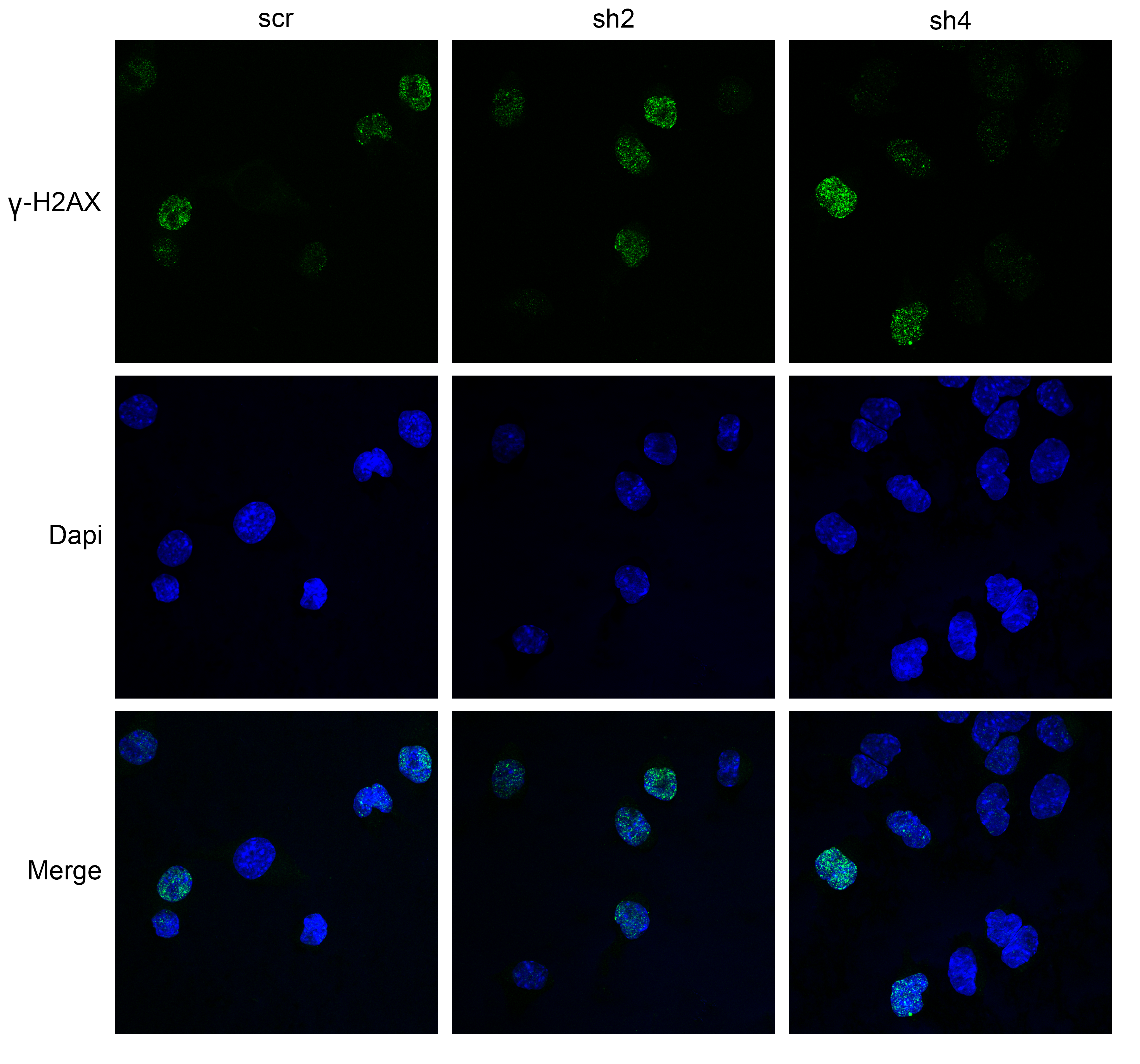

Supplement: S7 Fig — Immunofluorescence staining of γ-H2AX in Mvt1 cells with Rnaseh2c knockdown. Cells were grown to approximately 50% confluency on glass coverslips for staining. One of two independent experiments is shown. Magnification, 63X. (TIF) [file pgen.1008020.s007.tif]

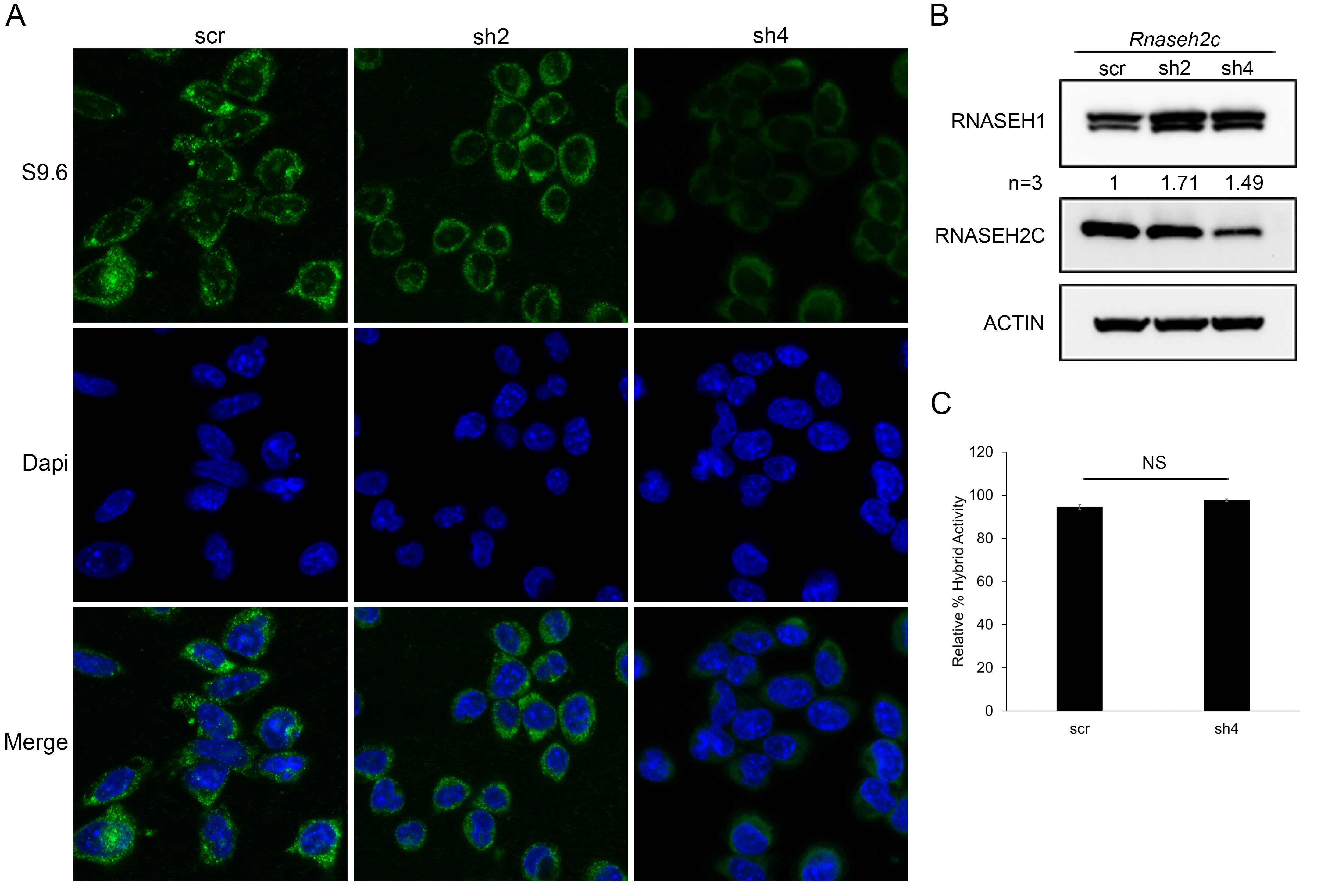

Supplement: S8 Fig — (A) Immunofluorescence staining of RNA/DNA hybrids using the S9.6 antibody in Mvt1 cells with Rnaseh2c knockdown. One of three independent experiments is shown. Magnification 100X. (B) RNASEH1 protein expression upon Rnaseh2c knockdown. Densitometry relative to Actin for three independent experiments is reported below. (C) Percent RNA/DNA hybrid (RNase H) activity in Mvt1 cells with knockdown of Rnaseh2c. Activity levels are presented relative to untransduced Mvt1 cells. Average ± standard deviation of two technical replicates. NS—not significant. (TIF) [file pgen.1008020.s008.tif]

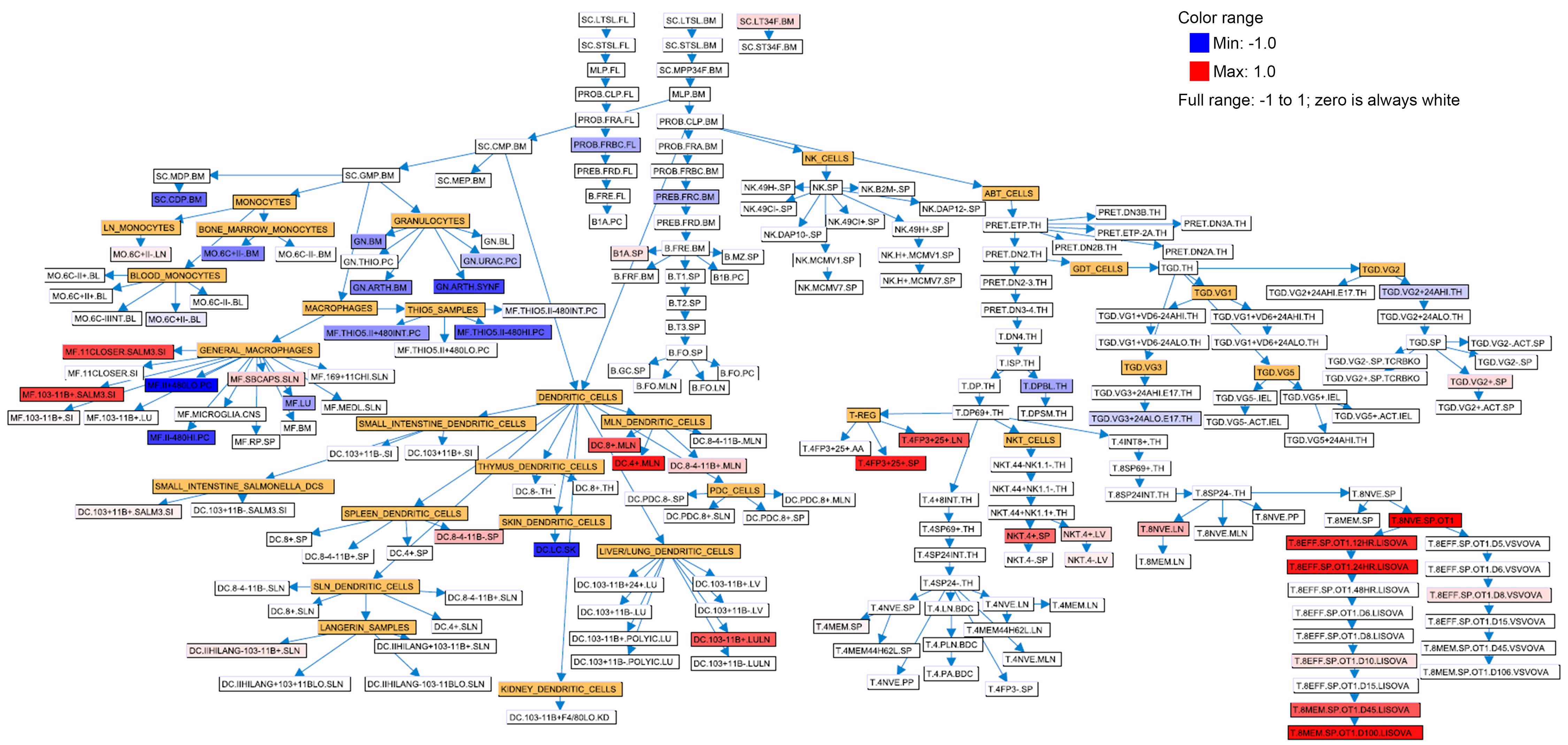

Supplement: S9 Fig — mRNA-sequencing data was analyzed using ImmQuant software for changes in immune cell-specific gene expression and compared to reference gene expression profiles from defined inflammatory states. Predicted presence of immune cell types identified in the sh4 tumors are reported between -1 (dark blue, lowest presence) and 1 (dark red, highest presence) compared to scramble control tumors. Categories of immune cells are shown in yellow. (TIF) [file pgen.1008020.s009.tif]

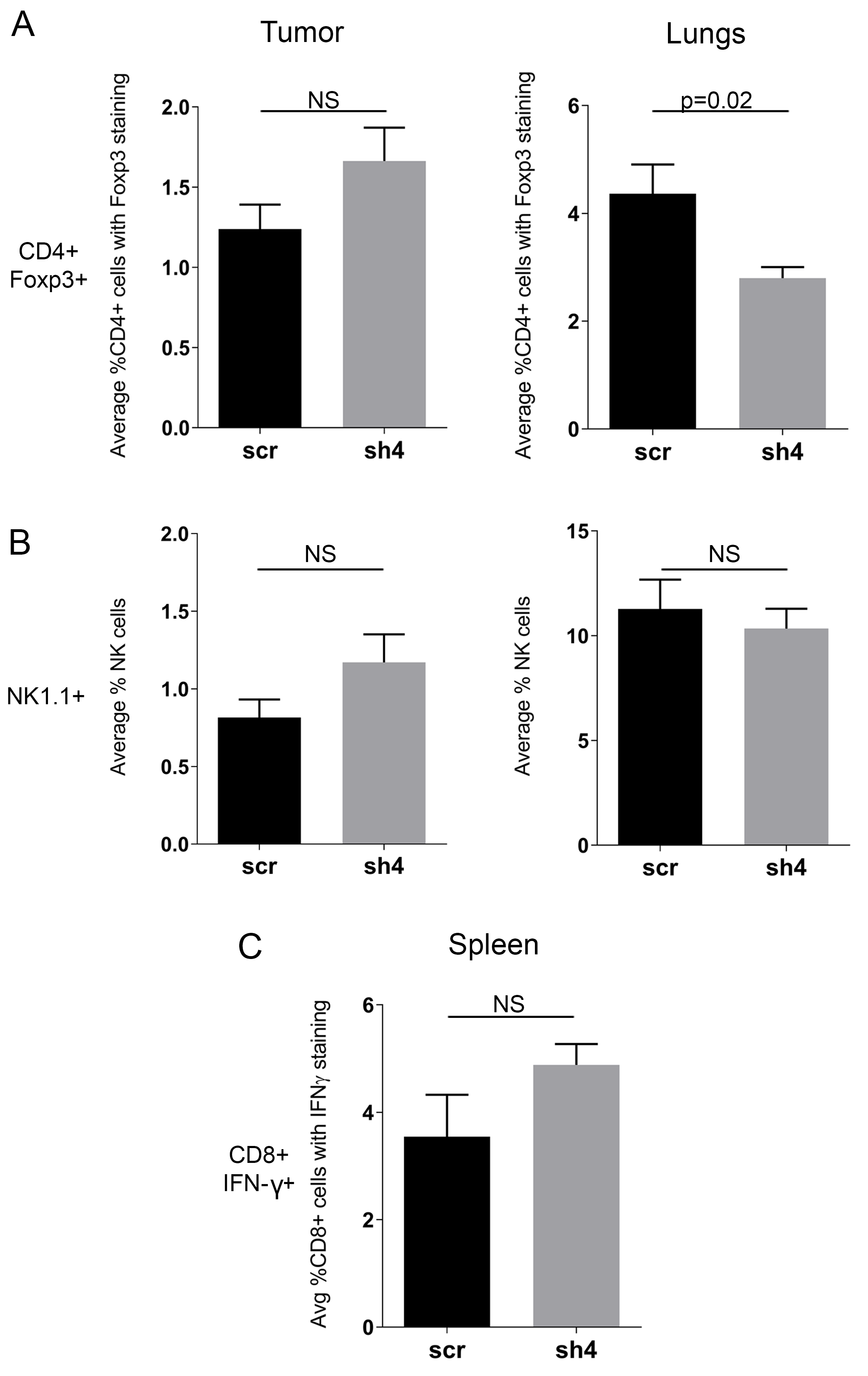

Supplement: S10 Fig — Immunophenotyping of cells within the primary tumor (left) or metastatic lungs (right) at euthanasia: (A) Average percent T regulatory cells identified by CD4+ Foxp3+ staining. (B) Average percent natural killer (NK) cells identified by NK1.1 staining. (C) Presence of activated (IFN-γ producing) CD8+ T cells in the spleen at euthanasia. Average ± SEM; NS—not significant. (TIF) [file pgen.1008020.s010.tif]

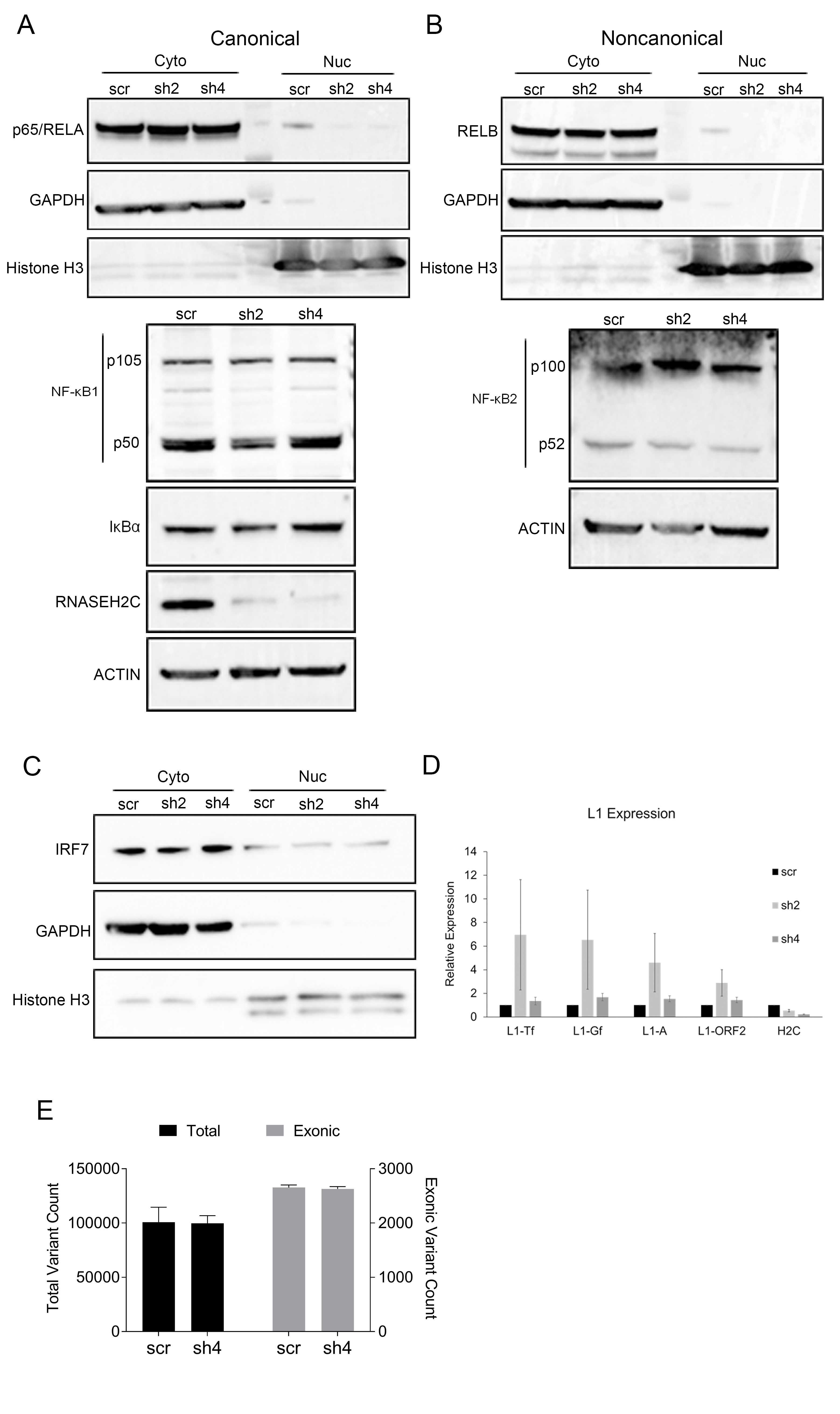

Supplement: S11 Fig — (A) Western blot analysis of canonical NF-κB signaling using fractionated (top) and whole cell (bottom) lysate from Rnaseh2c knockdown cells. (B) Western blot analysis of noncanonical NF-κB signaling using fractionated (top) and whole cell (bottom) lysate from Rnaseh2c knockdown cells. (C) Western blot analysis of IRF7 nuclear translocation in the Rnaseh2c knockdown cells following fractionation of the cytoplasmic (Cyto) and nuclear (Nuc) fractions. (D) Analysis of three families of L1 elements by qRT-PCR. Average ± standard deviation of three experiments. (E) Exome sequencing to analyze mutation burden (number of sequence variants) following Rnaseh2c knockdown. Average ± standard deviation; n = 4 metastases per group. (TIF) [file pgen.1008020.s011.tif]

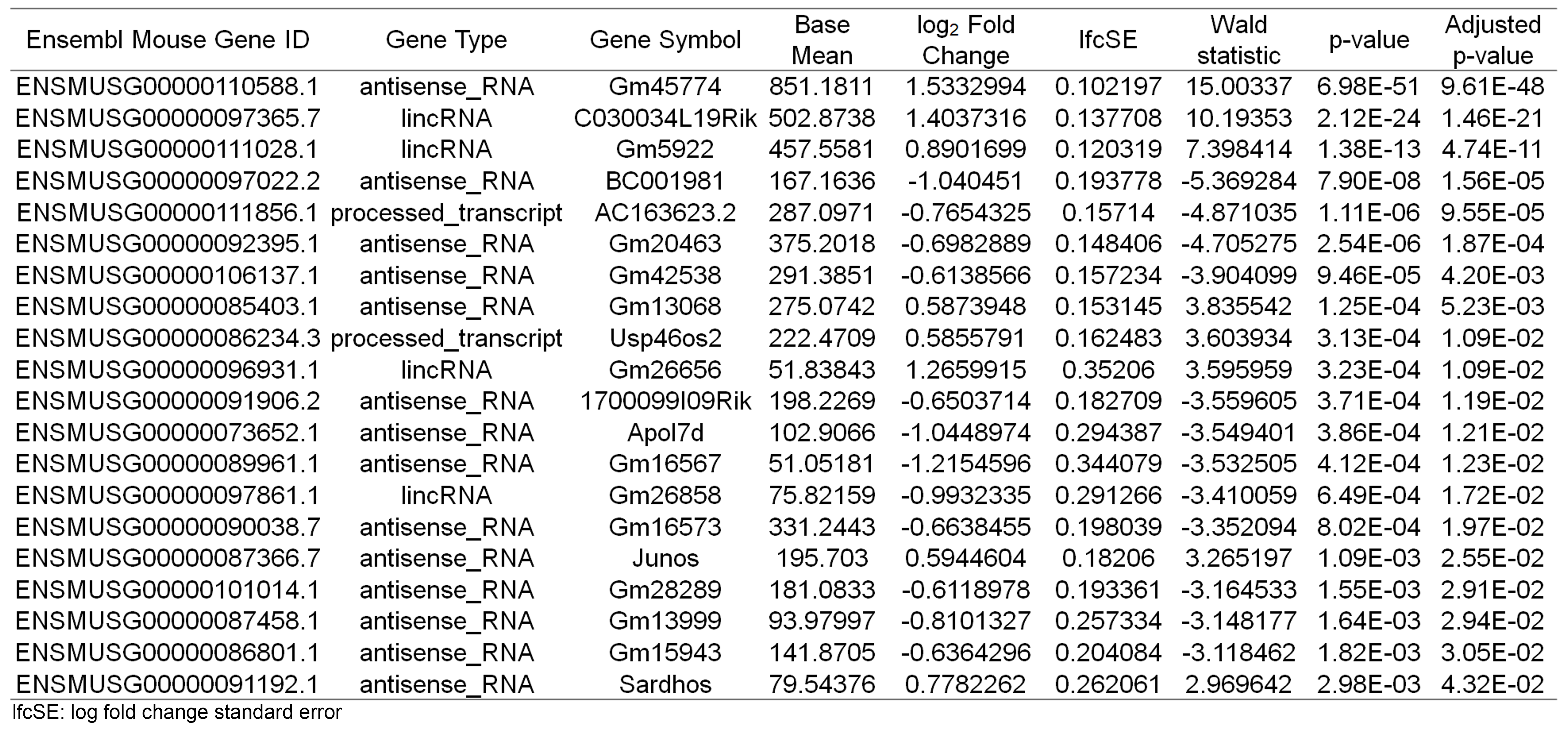

Supplement: S1 Table — (TIF) [file pgen.1008020.s013.tif]

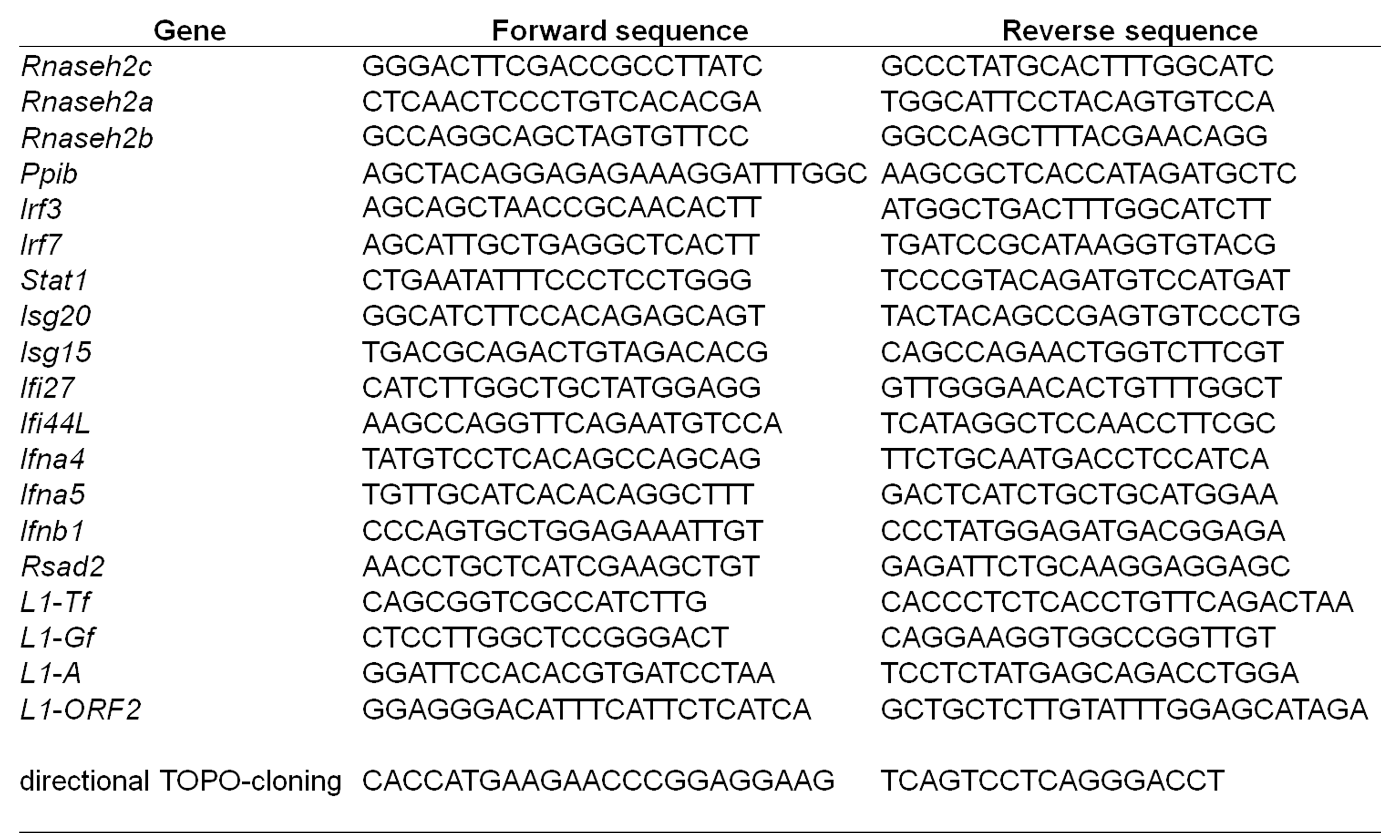

Supplement: S2 Table — (TIF) [file pgen.1008020.s014.tif]
